# Depth of field of multi‐slice electron ptychography: Investigating energy and convergence angle

**DOI:** 10.1111/jmi.70039

**Published:** 2025-10-06

**Authors:** Frederick Allars, Andrew Maiden, Darren J. Batey, Christopher S. Allen

**Affiliations:** ^1^ Electron Physical Science Imaging Centre Diamond Light Source, Harwell Science and Innovation Campus, Fermi Avenue Didcot UK; ^2^ Diamond Light Source Harwell Science and Innovation Campus, Fermi Avenue Didcot UK; ^3^ School of Electrical and Electronic Engineering University of Sheffield Sheffield UK; ^4^ Department of Materials University of Oxford Oxford UK

**Keywords:** multi‐slice, ptychography, transmission electron microscopy, 4D‐STEM

## Abstract

Multi‐slice electron ptychography has attracted significant interest in recent years, thanks to notable experimental successes in ultra‐high resolution, depth‐resolved imaging of atomic structure. However, the theoretical dependence of depth of field on experimental parameters is not well understood. In this paper we use simulated data to compare the depth of field of through focal annular‐dark field and multi‐slice electron ptychography over a range of acceleration voltages and convergence angles. We show that at both low convergence angle and at low electron energy, multi‐slice ptychography has significantly improved depth of field over through focal ADF imaging.

## 1 INTRODUCTION

Conventional transmission electron microscope (TEM)‐based atomic resolution imaging techniques provide two‐dimensional projection images of the sample. The ability to resolve structure along the incident beam (axial) direction, however, is critical for the understanding of three‐dimensional structures such as dislocations, dopants and grain‐boundaries which can have a significant impact on the functional properties of materials. Numerous methods have been developed to access this information, including electron tomography, annular dark field through focal series (ADF) imaging and confocal imaging. These methods have been used in a variety of applications, for example, to locate heavy dopants within crystal structures of lower atomic number,^1^ image Pt nanowires within environmental cells^2^ and remove the effect of silicon windows in in situ cell experiments.^3^, ^4^ The depth of field (DoF) of these techniques determines their axial resolution, and is itself determined by the illumination conditions of the microscope. With the advent of high‐order aberration correctors,^5^ the DoF of ADF imaging has significantly improved, reaching 2 nm at a convergence angle of 70 mrad.^6^ Electron tomographic reconstructions have gone further, achieving atomic resolution three‐dimensional imaging,^7^, ^8^ but collecting images across a range of tilt angles requires relatively long acquisition times and a high cumulative electron dose.^9^ With the advent of multi‐slice electron ptychography (MEP) – where multiple elastic scattering within the sample is compensated by multi‐slice modelling of the electron beam, as originally outlined by Cowley and Moodie^10^ – it is possible to achieve a DoF beyond the limits of ADF, even for thicker samples. The first demonstration of multi‐slice electron ptychography was performed by Gao et al.,^11^ where they demonstrated the separation in depth of the two overlapping carbon nano tubes via the application of multi‐slice electron ptychography. To date, samples as thick as 40 nm having been investigated with MEP^12^, ^13^ and DoFs down to 3.9 nm have been achieved.^14^

In ADF imaging, both lateral resolution and DoF are determined by the size of the focussed electron beam at the specimen, which governs the volume of the specimen that most strongly contributes to the detected signal. The beam convergence angle determines the size of the focused beam, and the accurate control of lens aberrations made possible by modern aberration correction now means convergence semi‐angles of more than 60 mrad can be achieved,^15^, ^16^, ^17^

The DoF of ADF imaging, $d_{\alpha }$, is defined by Equation (1):^5^, ^16^

(1) $$d_{\alpha }=\sqrt{{\left(\frac{d_{0}\lambda }{\alpha^{2}}\right)}^{2}+{\left(\frac{C_{C}\Delta E}{E_{0}}\right)}^{2}},$$

where $\;d_{0}$ is an empirical constant with values between 1 and 2,^16^ λ is the electron wavelength, α is the convergence semi‐angle, $\;C_{C}$ is the chromatic aberration coefficient, and $\;\Delta E/E_{0}$ is the spread in acceleration voltage as a fraction of the mean acceleration voltage.

In contrast to ADF imaging, neither the lateral resolution nor the DoF of multi‐slice ptychography are limited by the illumination convergence semi‐angle but are instead constrained by the maximum angle at which there is significant signal collected by the detector.^14^

A ptychographic experiment involves collecting a four‐dimensional scanning‐TEM (4DSTEM) data set with a high‐speed pixelated detector (ensuring overlap of the illuminated regions of the specimen between scanning positions) and then feeding this 4D dataset into reconstruction algorithms to recover a phase image of the specimen.^18^, ^19^, ^20^, ^21^, ^22^ Multi‐slice ptychography extends this reconstruction process to account for thick specimens where multiple elastic scattering occurs. A detailed description of the multi‐slice algorithm can be found, for example, in the work of Maiden et al.,^23^ Chen et al.^14^, ^22^ and Varnavides et al.^24^

The highest lateral resolution, $r_{x}$, in a ptychographic reconstruction corresponds to the case where a measurable signal is recorded right to the edges of the detector, and is given by Equation (2):

(2) $$r_{x}=\frac{\lambda }{\beta }=\frac{2\lambda C_{l}}{U\Delta u},$$

With 𝛽 being the angle subtended by the detector from its centre to its edge, $C_{l}$ is the camera length, U the number of pixels in each dimension of the detector and Δu is the physical size of the detector pixels.

The DoF of single slice electron ptychography, D, is then given by Equation (3):^25^

(3) $$D\leq \frac{c{\left(r_{x}\right)}^{2}}{\lambda }=\frac{c\lambda }{\beta^{2}},$$

**FIGURE 1:**
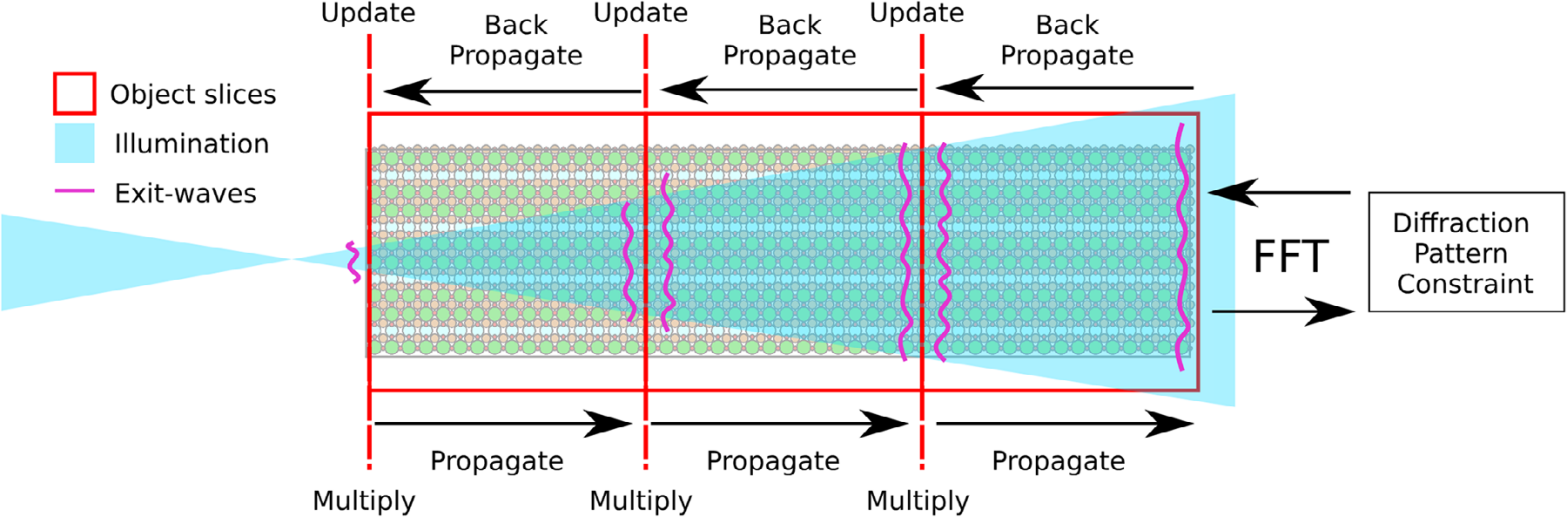
An illustration of the multi‐slice ptychographic reconstruction process.

Where c is an empirical constant discussed in the supplementary material of Refs. (25) and (26). Equation (3) is the latest iteration of equation defining the DoF of ptychography, other examples include Thibault et al. who in the supplementary material of Ref. (27) set out the derivation the DoF of ptychography from the homogeneous time‐independent wave equation. Samples thicker than DoF outlined by Equation (3) cause image artefacts, ranging from diffractive ‘ringing’ around features through to complete failure of the algorithms to converge. Multi‐slice ptychography enables image reconstruction from samples thicker than the conventional limit imposed by Equation (3). The multi‐slice model splits the sample volume into a series of slices, with each slice separated from the next by a distance less than the DoF, as shown in Figure 1. Reconstruction algorithms then incorporate this model to reconstruct a series of slice images that computationally section the thick sample, with a lateral resolution of $r_{x}$ (in theory) maintained for each image slice, even for samples several 10s of nanometres thick. Assuming Equation (3) is applicable to electron ptychography, with a suitable value chosen *c*, Equation (3) suggests that a DoF for MEP can be superior to that of through focal ADF when the pixelated detector subtends an angle greater than the convergence semi‐angle (α<β) as we will show in the simulation results that follow.

In this paper we describe a method to assess the axial resolution of MEP and test, using simulated data generated by the abTEM software,^28^ the accuracy and applicability of the limit given by Equation (3). We show that MEP beats the DoF limitations which constrain ADF, yet that it too can benefit from aberration corrected optics, especially at low electron dose.

## 2 METHOD

To assess the DoF of multi‐slice electron ptychography, we simulate 4DSTEM and ADF data sets from an 8.2 nm thick sample of Cerium‐doped hexagonal Boron nitride (h‐BN) using the abTEM python module.^28^ The scan step size in the ADF and ptychography simulations are both set to 0.195Å, which ensures a sufficient degree of overlap for the ptychographic reconstruction.

For the ADF simulation, a series of images corresponding to different beam foci are simulated, with a simulated detector collection range spanning 55 to 120 mrad. The through focal series starts at 6.5 nm above and ends 7.3 nm below the bottom surface of the simulated samples with an axial step size of 0.5 nm.

The 4DSTEM data is simulated with a positive beam defocus to ensure that the probe size – and therefore the degree of overlap between beam scan positions – increases as the illumination propagates through the specimen. The degree of defocus was chosen such that when the beam first arrives at the specimen the size of the probe was approximately equal for all convergence angles. Frozen phonons where included in the simulations using abTEM's in build frozen phonon modelling, using 4 different configurations and the standard deviation in atomic displacement between configurations being 0.15 Å. The update parameters are the same for all reconstructions to minimise the parameter space being investigated. In this case the probe update step size^29^ was chosen to be 0.4 and the object update step size was 0.05. Diffraction patterns were generated from a detector that subtends a half angle of 120mrad in the *x*‐ and *y*‐dimensions. The detector consisted of 416 × 416 pixels at 300 KeV, 424 × 424 pixels at 200 keV and 430 × 430 pixels at 80 KeV, with a corresponding reconstructed image pixel size of 8.2 pm, 10.4 pm and 18.2 pm respectively. The number of detector pixels used in the simulation varies slightly between energies to maintain a constant frequency space sampling across all simulations. Following Equation (3), a detector subtending 120 mrad should enable a DoF of 7.11 Å at 300 KeV, 9.06 Å nm at 200 KeV and 15.08 Å at 80 KeV beam energies (assuming *c* of 5.2).

Reconstructions were performed using the ePIE^30^ implementation in PtyREX, a ptychographic reconstruction package developed by the Diamond Light Source,^31^ adapted for multi‐slice in the manner described by Maiden et al.^23^ A further modification included the multi‐slice back‐propagation method outlined by Varnavides et al.,^24^ while further constraints were applied to the ptychographic reconstruction^32^ – a fixed image magnitude of 1 and clamping of negative phases.

**FIGURE 2:**
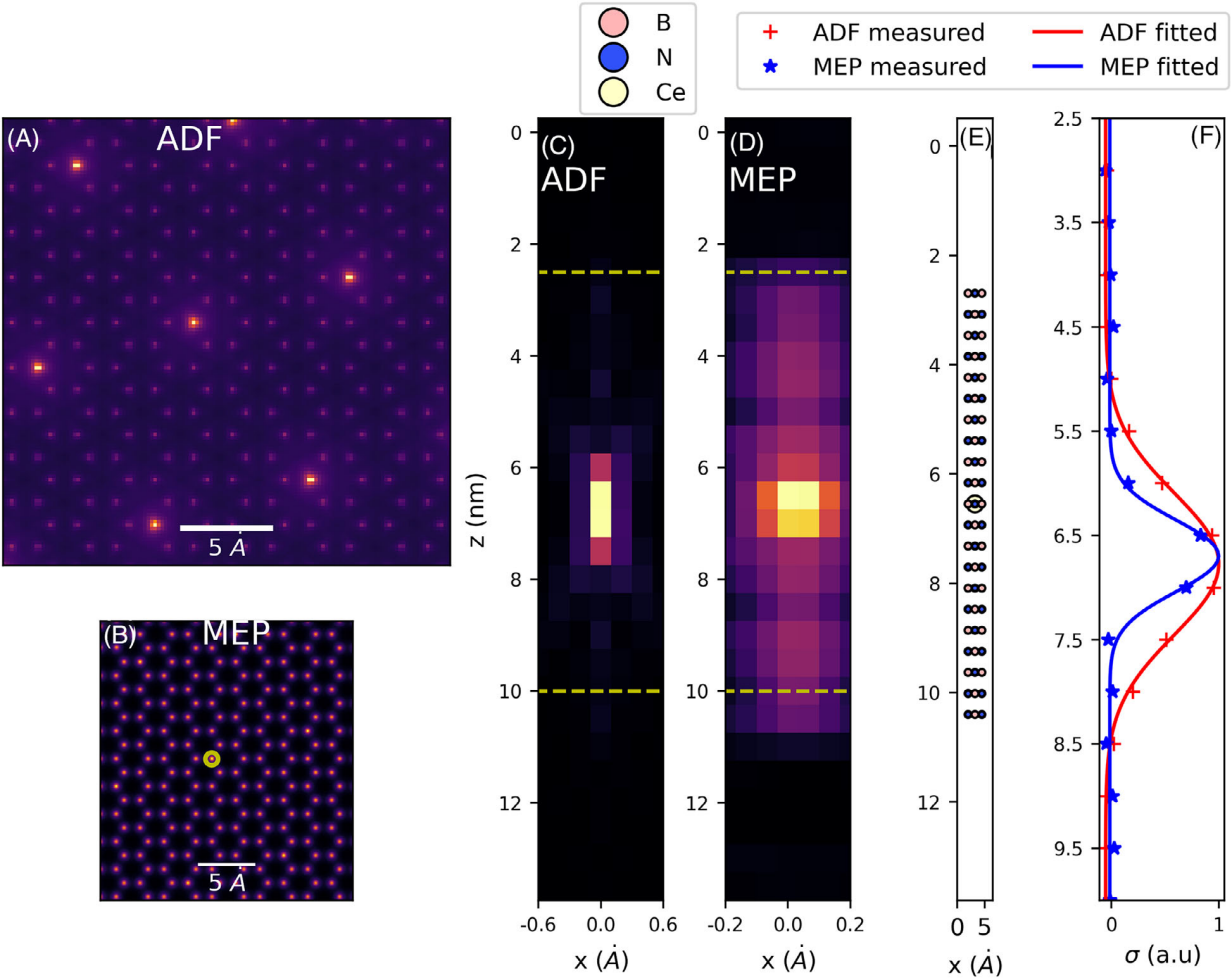
(A) Summed Intensity from simulated ADF of BN doped with Ce atoms (contrast adjusted). (B) Sum of the phase slices from MEP with a doped column highlighted with yellow circle. (C, D) *x‐*‐*z* slice at the doped column highlighted in (B) for the ADF and MEP slices respectively. (E) A subsection of the abTEM model showing a doped column with a Ce atom, this model was used to generate ptychographic data and ADF images. (F) A plot of the normalised standard deviation in the *x*–*y* plane as a function of depth for the ADF and MEP slices respectively from the area highlighted by dashed yellow lines in (C) and (D).

The ADF and ptychography simulations were processed by first creating a sum of the z‐stack of images. (In the case of multi‐slice ptychography, it is the phases of each object slice that are summed.) The Atomap python package was then used to identify the positions of all atomic columns^33^ Individual atomic columns were isolated by selecting a square area around the column centre with a width of 5 pixels in the *xy* plane. For each atomic column in the ADF through focal series the standard deviation in the *xy* plane was calculated following the process outlined by Ishikawa et al.,^34^ which is plotted as a function of depth (*z*) in Figure 2E.

As multi‐slice ptychography is a phase sensitive technique, the BN lattice within the Ce‐containing slice contributes a significant phase shift to the reconstruction, which is added to the phase of the Ce atom. As ADF contrast is a power law in atomic number (*Z*), however, the BN lattice contribution to the Ce intensity in the ADF through focal series is minimal (see Figure S1).

To measure DoF, the average phase of the BN lattice was first measured and subtracted from the Ce‐doped column profile to isolate the signal from the dopant atom. An example of the substrate subtraction can also be seen in Supplementary Material (SF 1). The standard deviation of the signal in the *xy* plane is then calculated, allowing a direct comparison between our results and those from the work of Ishikawa et al.^34^

The DoF was measured by fitting a Gaussian function (Equation 4) to the standard deviation depth profile of each doped column in turn (there are seven within the simulated field of view; see Figure 2A), with the full width half maximum (FWHM ≈2.355σ) of these fits are then averaged across all of the doped columns used as a measure of the DoF.

(4) $$y=A\left(\exp\left(\frac{-{\left(z-p\right)}^{2}}{2\sigma^{2}}\right)\right)+b$$

Here y is the curve to be fitted to standard deviation/depth profile, A is the amplitude of the gaussian, z is the displacement in depth, p is the centre of the Gaussian, σ is the standard deviation of the gaussian, and b is offset to account for any offset in the recovered phase.

## 3 RESULTS

### 3.1 Beam energy and convergence angle dependence of the depth of field

**FIGURE 3:**
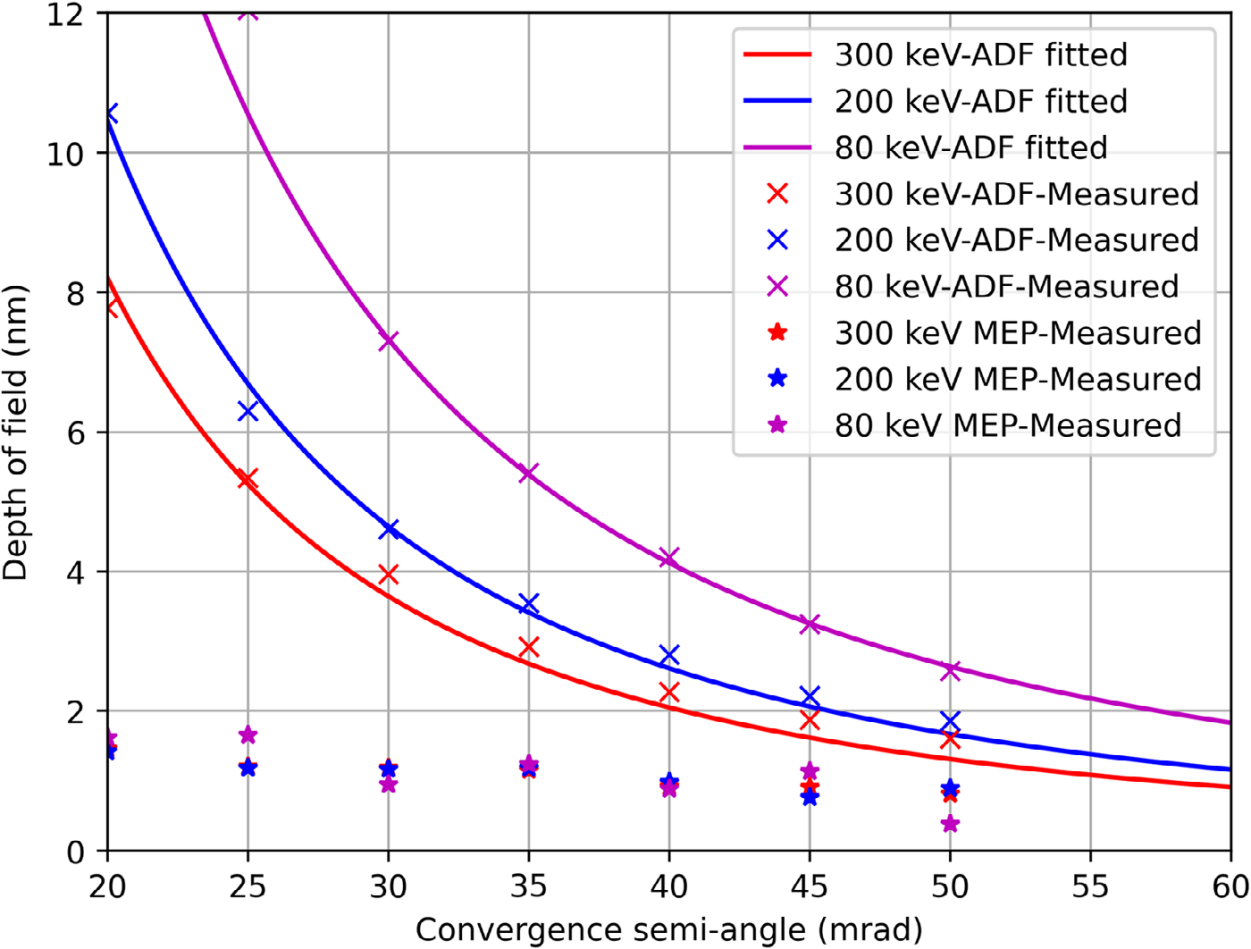
A comparison of simulated DoF of ADF and MEP over a range of energies and convergence angles.

Figure 3 shows the DoF, measured as described above, resulting from simulated data sets across a range of beam energies and convergence semi‐angles. For multi‐slice ptychography, the DoF is decoupled from the limitations defined by the optics of the illumination system. Furthermore, the dependence on beam energy is significantly reduced compared to the ADF simulations suggesting that MEP is capable of a high DoF even at low beam energies.

It is worth noting that, although the DoF of MEP has a weak dependence on the convergence angle, there remains a slight decrease in resolved depth at lower convergence angles. This is likely due to the high but finite dose in the simulation: $10^{8}$ e/Å^2^, which leads to low counts and signal‐to‐noise at high diffraction angles for the low convergence angle data. Nevertheless, these simulation results confirm that the convergence angle has little effect on the DoF of MEP, in contrast to ADF imaging. The achieved DoF at the higher convergence angles for MEP is better than the theoretical prediction from Equation (3) especially for the 80 keV results; this brings into question the value chosen for the empirical constant *c* (*c* = 5.2) in Equation (3), which may not be suitable for the electron domain.

### 3.2 Dose dependence of the depth of field

To determine the dependence of the DoF on electron dose for MEP, a second simulation study was performed. The same Ce‐doped BN structure was studied at 200 KeV over a range of convergence angles, with electron dose varied from $10^{5\;}\;\mathrm{to}\;10^{7}$ e/Å^2^ and the DoF was measured in the manner previously discussed. The variation in dose was implemented using the Poisson noise model within abTEM.^28^ Lattice vibrations have been accounted for in simulations results shown in Figure 5 via abTEM frozen‐phonon model. We do not observe significant change in the DoF when including the frozen phonons in the simulation. This is likely due to lateral resolution of the reconstructions not being sufficiently high to be affected by atomic thermal vibrations.

**FIGURE 4:**
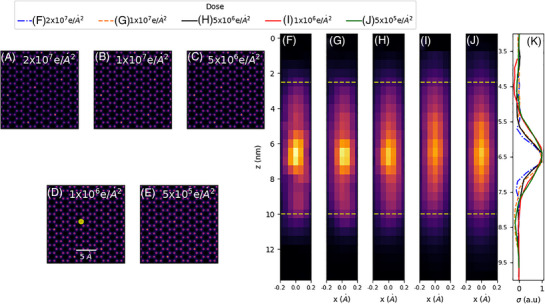
Dose dependence of the DoF of MEP at 30 mrad semi‐convergence angle. (A–E) A single slice of the MEP reconstruction showing strong Ce atom contrast and (F–J) *x*–*z* slice at the doped column highlighted in (D) at doses of $2\times 10^{7}$(A, F), $1\times 10^{7}$(B, G), $5\times 10^{6}$(C, H), $1\times 10^{6}e/{Å}^{2}$(D, I), and $5\times 10^{5}$(E, J). (K) The standard deviation of Ce atom intensity in the *x‐*‐*y* plane as a function of depth.

Figure 4F–J shows the effect of electron dose on the DoF of MEP. As electron dose decreases there is a blurring of the Ce atom in the *z*‐direction from a FWHM of 1.52 nm at $2\times 10^{7}$ e/A^2^ to 3.08 nm at $5\;\times \;10^{5}$ e/A^2^ when the convergence angle is 30mrad. The lateral resolution of the reconstruction remains largely unchanged across this dose range. This decrease in DoF can be attributed to two factors. Firstly, multiple scattering within the sample increases the high angle scatter in the emerging exit wavefront, and thus improves DoF. As the number of electrons scattered to high angle is relatively small, at low electron dose this contribution is reduced. Secondly, MEP is attempting to solve for the phase and amplitude of the incident probe function and object transmission function of each slice. To do this robustly requires a sufficiently large number of measurements in the data set and therefore the ptychographic inversion is less well conditioned at lower dose.

**FIGURE 5:**
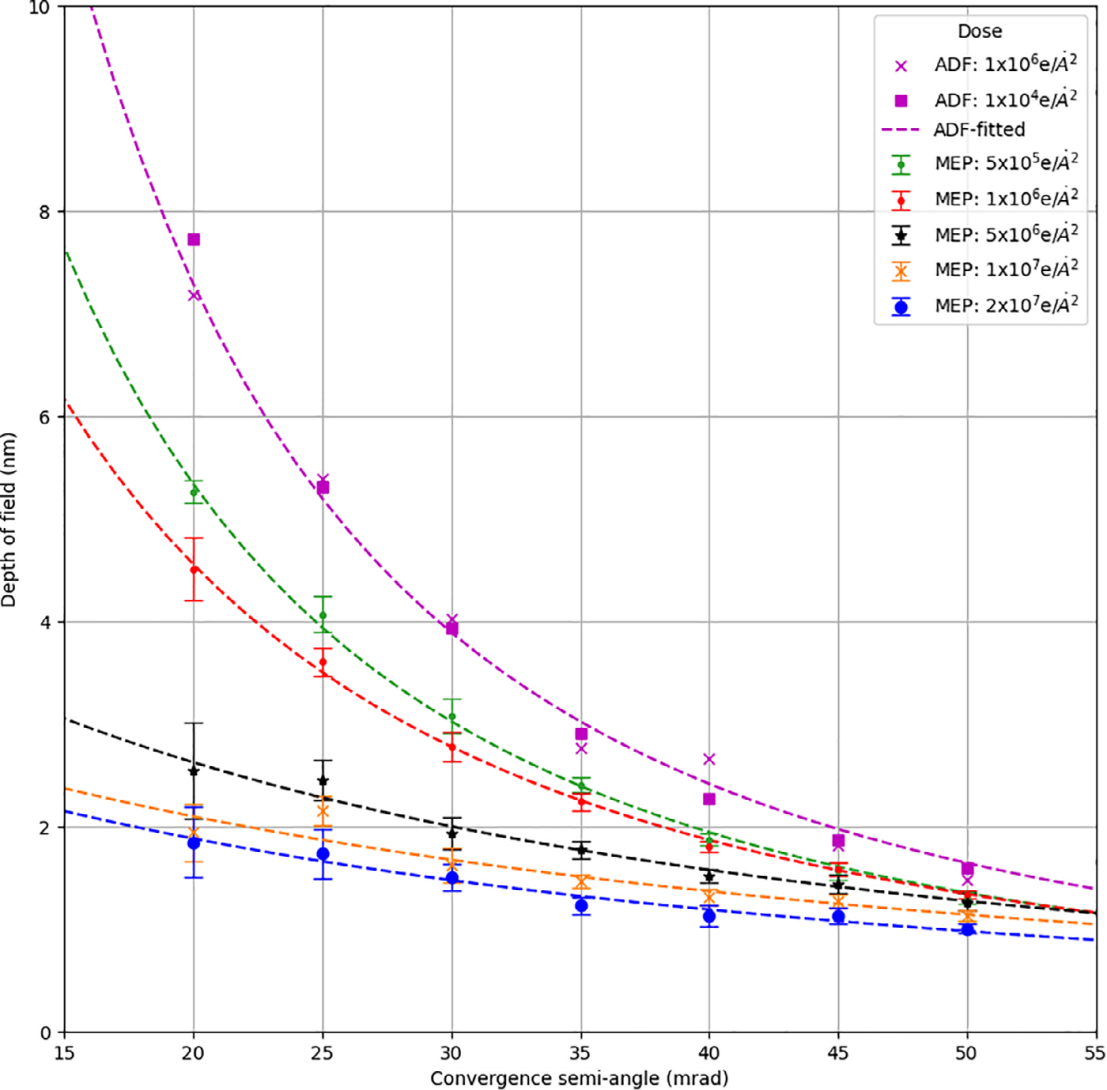
DoF of simulated ADF and MEP reconstructions as a function of electron dose and convergence semi‐angle for 300 keV acceleration voltage. The dot markers correspond to average depth measurements from simulated data, the error bars indicate the variance in DoF measured from different atomic columns, and dotted lines are guides for the eye.

Figure 5 shows the measured DoF of MEP as a function of electron dose and convergence semi‐angle as measured from simulated data at 300 keV. As expected, the DoF of the reconstructed MEP image decreases as the incident electron dose decreases. The reduction in DoF between MEP measurements at lower doses is less significant as the convergence angle increases, suggesting that for optimal DoF at low dose a higher convergence semi‐angle is preferable. For reference, measurements of the DoF of 300 KeV ADF at doses of 10^4^ and 10^6^ are added to show that this is largely independent of dose within reasonable experimental values. Direct comparison between ADF and MEP is somewhat complex due to the different data collection methods (i.e. probe overlap and number of measurements). Furthermore, simulation studies have shown that MEP reconstructions are possible at electron doses as low as $10^{4}$ e/Å^2^.^14^

## 4 DISCUSSION

The DoF of ADF imaging is governed by the volume within the specimen that is strongly illuminated by the beam, which is determined by the beam convergence angle. The situation is less straightforward in MEP. The coherent scatter occurring within the specimen structures the beam, such that it collects higher spatial frequencies during its passage through the sample. These high spatial frequencies change significantly when propagated between slices in the model, enabling their separation by the MEP algorithm and the increase in DoF we observe compared to ADF imaging. The higher spatial frequencies can be considered as an effective increase in the convergence angle of the MEP technique beyond what is available to ADF. However, if the specimen is weakly scattering and does not induce sufficient high frequency structure outside of the bright field disc, the DoF will remain limited by the convergence semi‐angle of illumination. Lastly it is common practice in ptychographic reconstructions to padded diffraction patterns such high spatial frequencies can be recovered, by allowing the solution of these padded pixels to float during the intensity update.^35^, ^36^ This in theory should allow for improved DoF compared to non‐padded data and therefore requires investigation in tandem with variations in the dose and convergence angle.

The values for DoF we obtain from our simulations are worst that those estimated using Equation (3). For example, Equation (3) gives DoF values of 7.11 Å at 300 KeV, 9.06 Å at 200 KeV and 15.08 Å at 80 KeV, whereas even at high convergence angles our simulations do not achieve a DoF better than approximately 15 Å. This illustrates that care must be taken in the use of Equation (3) to approximate DoF for MEP, as the parameter *c* is poorly defined and the effects of experimental and reconstruction parameters such as electron dose and reconstruction slice thickness are not accounted for.

Our results suggest that MEP can provide superior DoF than ADF imaging in most situations. Unlike ADF, MEP also obtains all the required data from a single *xy* scan, meaning that it is more convenient experimentally, avoiding the need to correct for aberrations mid‐experiment and to cross‐correlate results post data collection. Moreover, some specimens can be altered when imaged under the electron beam; MEP simplifies these experiments as the slices should be consistent while different focal planes in ADF may no longer fully correlate due to specimen damage. The ongoing improvements to aberration correction in STEMs will provide an improved DoF in both ADF^5^ and MEP imaging, in the case of MEP allowing for lower dose experiments in the future. Figure 5 suggest that for current electron microscopes, which typically use a convergence angle of up to 30 mrad, the most important factor limiting DoF is the amount of dose applied. Although higher collection angles do improve DoF in MEP, the quadratic relation given in Equation (3) implies diminishing returns, and there is still an incentive to pursue electron ptychographic tomography.^37^, ^38^

The independence of both lateral resolution and DoF from beam convergence angle (the lateral case has already been discussed in Ref. 39) allows us to design experiments with greater freedom. For example, one of the issues with large convergence angles is that the rate at which the beam changes can mean that the real space illumination function can become poorly sampled, causing it to ‘wrap around’ in the calculation window and thus limit sample thickness. This problem is alleviated if the large convergence angle can instead be traded for an increased dose at a lower convergence angle, while retaining the same DoF. Designing ptychographic experiments to obtain a set DoF is more involved than ADF, as the tools to determine a priori the experimental requirements for a set number of slices of desired thickness are not fully developed; currently, the data must be reconstructed before the DoF can be determined. Furthermore, as has been suggested above, the DoF for MEP is dependent on the specimen itself, not only on the optics of the microscope.

It is important to note that the work we present here is a simulation study. Aspects of experimental data which have not been considered in the simulations are higher‐order aberrations, thermal vibrations of the atomic structure,^40^, ^41^, ^42^ the effect of amorphous layers typical of FIB milled specimens, ptychographic sampling,^43^ inelastic scatter and the average atomic number of the atomic columns. While in Supplementary Material of this paper, there has brief study into the effect of atomic number of the dopant itself on the measured DoF which agrees with Chen et al. work^14^ in that as the atomic number of the dopant increases, the measured DoF reduces (see Figure S4).

Using a mixed‐state probe within forward model has already been shown to be vital to obtaining high resolution 3D reconstructions,^14^ but the effect of temporal incoherence on MEP is less well understood. The chromatic aberrations present in electron optics has been partially considered in Supplementary Material of this work (see Figure S3), and it has been shown that if left unaccounted for in the forward model cause temporal incoherence causes degradation in the DoF compared to coherent data. Further simulations are required to investigate the effect as degree of chromatic aberration changes and whether once the forward model has been modified to account for temporal incoherence the DoF recovers.

Furthermore, there are currently differing methods for measuring the DoF reported in the literature,^14^, ^44^ in which an error function is fitted to the intensity along atomic columns and DoF defined as the full width at 80% of the maximum (FW80% = 1.355σ); therefore, some care must be taken when directly comparing the results from these two measurement methods. Differing reconstruction methods and tuning parameters may be important such as the use of three‐dimensional filtering functions, slice update rates and other hyperparamters, which have not been used in this work in order to reduce the total number of parameters to be explored.^34^

## 5 CONCLUSION

In this paper, simulation studies have been used to investigate how the DoF of MEP changes as a function of acceleration voltage, convergence semi‐angle and dose. A method via which the DoF can be measured has been detailed and compared to previous ADF DoF techniques. We have shown that the DoF in MEP is largely independent of the acceleration voltage and the convergence semi‐angle, if there is sufficient dose. However, once the dose is lowered, DoF approaches that of ADF, therefore, to optimise future MEP experiments, understanding the dose limitations of the sample will be critical. Future work will include the collection of experimental data over a range of convergence angles, acceleration voltages and dose conditions, using a range of different specimens in order to better characterise the influence of the sample itself on DoF.

## Supporting information



Supporting Information
