# Supplementary material for: Depth of field of multi‐slice electron ptychography: Investigating energy and convergence angle
Source: J Microsc. 2025 Oct 6;300(2):191–200. doi: 10.1111/jmi.70039 (PMC12523988; doi:10.1111/jmi.70039)
Supplement: Supplementary file 1 — Supporting Information [file JMI-300-191-s001.docx]

# Supplementary materials:

Isolating the Ce dopant signal


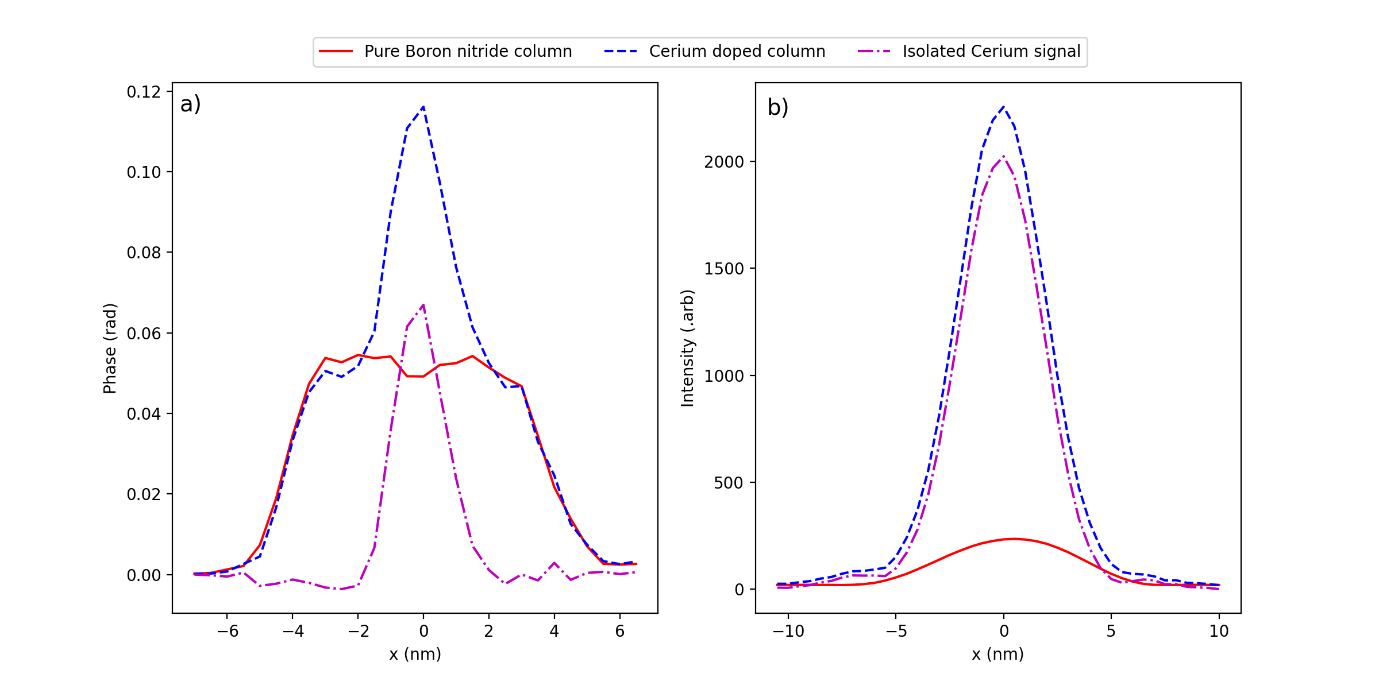


SF 1: The removal of the BN lattice signal from the Ce dopant atom signal in the case of a) the MEP reconstruction and b) the ADF through focal series.

Temporal Coherence and the Depth of Field of Multi-slice Electron Ptychography

Electron ptychography is fundamentally a coherent imaging technique and reconstructions are therefore degraded by any incoherence in the electron illumination. Spatial incoherence is routinely accounted for by including several incoherent modes in the illumination function [1]. For single slice ptychography the effect of temporal incoherence is generally considered to be negligible when using the Wigner Deconvolution method as this method accesses the achromatic lines within the double overlap region [2]. Iterative ptychography has been shown to be robust to chromatic aberrations at high convergence angles when investigating monolayer samples, however for thicker sample this still requires practical investigation [3], as the thinness of material may have minimised the effects of chromatic aberrations.

The simulated experimental conditions were identical to those used in section 4.1 of the main text, with the addition of temporal incoherence within the abTEM forward model, using inbuilt abTEM functions. Temporal incoherence was added to both ADF through focal series and MEP by simulating multiple diffraction patterns from a range of defocus values and weighting them in gaussian manner depending on their distance from the average defocus, before summing them incoherently. Focal values used had a mean of 4 nm and deviation of 0.25 nm, 11 samples were taken from this distribution (see figure 7), the samples were evenly spaced, relating to a $C_{c}=0.25 mm$ , $\Delta E$ = 0.3 eV at acceleration voltage of 300 KeV. This value of $C_{c}$ was chosen as it resulted in a measured $C_{c}=1.63 mm$, assuming a $\Delta E$ = 0.3. The value of $C_{c}$ was measured by fitting Equation 1 (main text) to the measured depth resolution of the ADF through focal simulation.


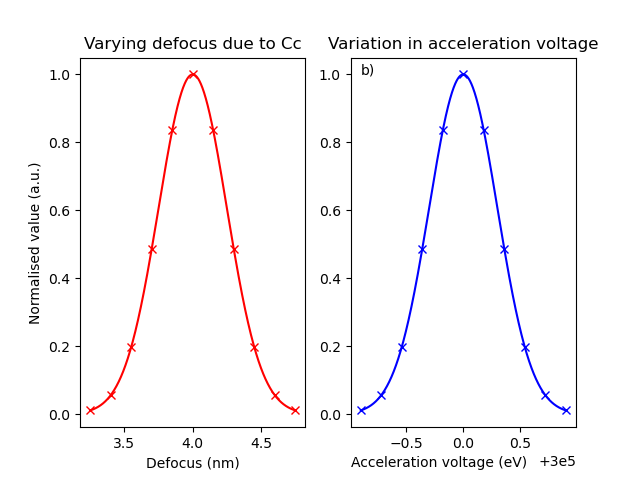


SF 2: a) A figure showing how the effective defocus and relative Intensity of simulated diffraction patterns was determined in order that they could be summed incoherently into a temporally incoherent data set. b) a figure showing how a) can be converted into the equivalent variance in acceleration voltage.


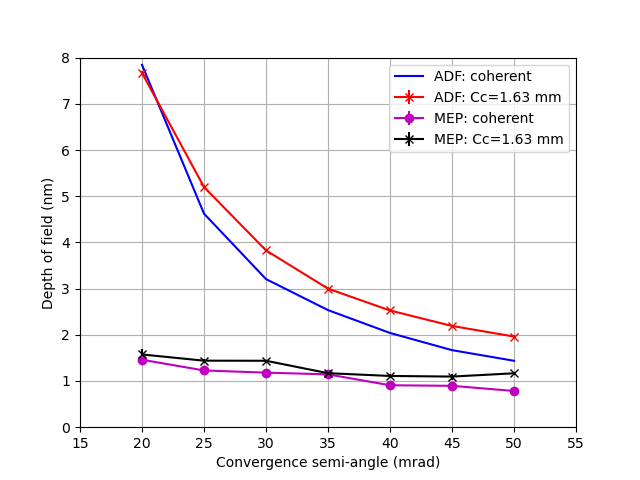


SF 3: Comparison between the measured DoF of simulated ADF and MEP reconstructions when the illumination is perfectly temporally coherent and partially incoherent.

Assuming a $C_{c}=1.63 mm$ applies to both MEP and ADF, SF 3 shows that the ADF DoF of our simulations is significantly degraded with the addition of temporal incoherence, in line with equation 1. Furthermore, it suggests that iterative MEP is worsened when considering temporally incoherent data, this is due to temporal incoherence not being accounted for in the forward model of the ptychographic reconstruction. This suggest that iterative MEP maybe be more susceptible to temporal incoherence than its analytical counterpart (Wigner Deconvolution). However, this requires confirmation with both further simulation studies and experimental investigation.

Effect of Atomic Number on the Depth of Field of Multi-Slice Electron Ptychography

In order to investigate effect of the atomic number on the measured DoF of MEP the same simulation parameters are used as in section 4.1 of the main text. The chosen acceleration voltage is 300KeV. Three dopants are investigated those being Cerium (Ce, 58), Copper (Cu, Z = 29) and Silicon (Si, Z = 14) which placed in identical positions to the Ce atom previously (see Figure 2 of the main text)


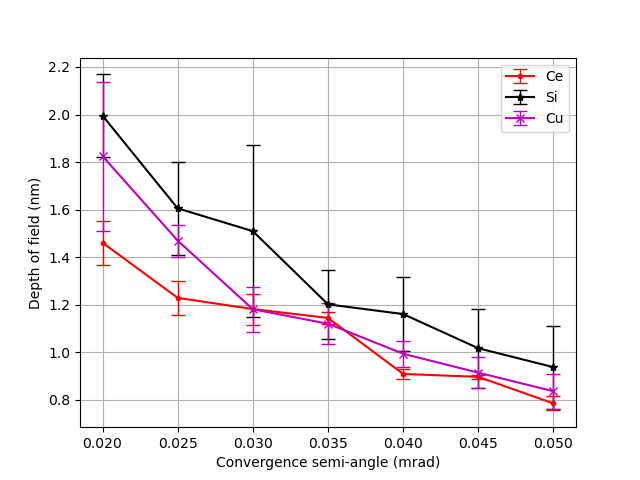


SF 4: A comparison between the DoF of different atomic number dopant atoms as the convergence angle changes. The dose for this simulation was set to $1\times{10}^{8}$ $e/Å^{2}.$

The results outlined in SF 4 find the same general trend found by Chen et al. in [4] where they noted that the different atomic columns had slightly different DoF. Chen et al. attributed this improved DoF to the greater contrast or the greater phase delay caused by the greater atomic number dopants.

References for the Supplementary materials

[1] P. Thibault and A. Menzel, “Reconstructing state mixtures from diffraction measurements,” *Nature*, vol. 494, no. 7435, pp. 68–71, 2013, doi: 10.1038/nature11806.

[2] P. D. Nellist and J. M. Rodenburg, “Beyond the conventional information limit: the relevant coherence function,” *Ultramicroscopy*, vol. 54, no. 1, pp. 61–74, 1994, doi: https://doi.org/10.1016/0304-3991(94)90092-2.

[3] Y. Ma, J. Shi, R. Guzman, A. Li, and W. Zhou, “Aberration Correction for Large-Angle Illumination Scanning Transmission Electron Microscopy by Using Iterative Electron Ptychography Algorithms,” *Microscopy and Microanalysis*, vol. 30, no. 2, pp. 226–235, Apr. 2024, doi: 10.1093/mam/ozae027.

[4] Z. Chen *et al.*, “Electron ptychography achieves atomic-resolution limits set by lattice vibrations,” *Science (1979)*, vol. 372, no. 6544, pp. 826–831, May 2021, doi: 10.1126/science.abg2533.
